# Trastuzumab deruxtecan in previously treated HER2‐positive metastatic or unresectable breast cancer: Real‐life data from the temporary use authorization program in France

**DOI:** 10.1002/cam4.7168

**Published:** 2024-05-11

**Authors:** Thierry Petit, Nawale Hajjaji, Eric‐Charles Antoine, Marc‐Antoine Benderra, Michel Gozy, Cyril Foa, Jean‐Loup Mouysset, Julien Grenier, Mireille Mousseau, Audrey Mailliez, Mahasti Saghatchian, Emma Lachaier, Isabelle Desmoulins, Audrey Hennequin, Patricia Maes, Delphine Loirat, Francesco Ricci, Véronique Diéras, Dominique Berton, Florence Lai Tiong, Luis Teixeira, Nadine Dohollou, Christelle Lévy, Thomas Bachelot, Jean‐Yves Pierga

**Affiliations:** ^1^ Département d'Oncologie Médicale Centre Paul Strauss Strasbourg France; ^2^ Département de Cancérologie Sénologique, Centre Oscar Lambret INSERM U1192, Laboratoire PRISM Lille France; ^3^ Département d'Oncologie Médicale Clinique Hartmann Neuilly‐sur‐Seine France; ^4^ Département d'Oncologie Médicale Hôpital Tenon Paris France; ^5^ Département de Radiothérapie‐Oncologie Clinique de l'Europe Amiens France; ^6^ Département d'Oncologie Médicale Hôpital Saint‐Joseph Marseille France; ^7^ Département de Cancérologie Hôpital Privé de Provence Aix‐en‐Provence France; ^8^ Unité Oncologie Sein‐Gynécologie Institut Sainte‐Catherine Avignon France; ^9^ Département d'Oncologie Médicale CHU Grenoble Grenoble France; ^10^ Département d'Oncologie Médicale Hôpital Américain de Paris Neuilly‐sur‐Seine France; ^11^ Département d'Oncologie Médicale CHU Amiens Amiens France; ^12^ Département d'Oncologie Médicale Centre Georges François Leclerc Dijon France; ^13^ Département d'Oncologie Hôpital Privé Le Bois Lille France; ^14^ Département d'Oncologie Médicale Institut Curie Paris France; ^15^ Département d'Oncologie Médicale Centre Eugène Marquis Rennes France; ^16^ Département d'Oncologie Médicale Institut de Cancérologie de l'Ouest Saint‐Herblain France; ^17^ Département d'Hémato‐Oncologie CHU Sud Réunion Saint‐Pierre France; ^18^ Breast Disease Unit, APHP, Hôpital Saint‐Louis, Pathophysiology of Breast Cancer Team Université de Paris, INSERM U976, HIPI Paris France; ^19^ Service d'Oncologie Médicale Polyclinique Bordeaux Nord Aquitaine Bordeaux France; ^20^ Département d'Oncologie Médicale Centre François Baclesse Caen France; ^21^ Département d'Oncologie Médicale Centre Léon Bérard Lyon France; ^22^ Université Paris Cité Paris France

**Keywords:** breast cancer, early‐access program, HER2‐positive, metastatic cancer, trastuzumab deruxtecan

## Abstract

**Background:**

Early access program (formerly cohort Temporary Authorization for Use) was granted for trastuzumab deruxtecan (T‐DXd) in France based on DESTINY‐Breast01 trial which demonstrated its efficacy and safety in HER2‐positive metastatic/unresectable breast cancer after ≥2 anti‐HER2‐based regimens received at metastatic stage.

**Method**s**:**

This multicenter real‐world early access program included HER2‐positive metastatic/unresectable breast patients pretreated with at least two lines of anti‐HER2 regimens who received T‐DXd 5.4 mg/kg intravenously in monotherapy every 3 weeks.

**Results:**

Four hundred and fifty‐nine patients (median age, 58 years; hormone receptor‐positive, 67%; brain metastases, 28.1%) received T‐DXd. Before inclusion, 81.7% of patients had radiation therapy and 76.5% had undergone surgery. Median number of prior metastatic treatment lines was four (range, 2–22); 99.8% patients had received trastuzumab, 94.8% trastuzumab emtansine and 79.3% pertuzumab. Follow‐up was performed from September 30, 2020 to March 30, 2021; when the early access program stopped, the median duration of T‐DXd treatment was 3.4 (range, 0–7.8) months. In 160 patients with available tumor assessment, objective response rate was 56.7% and 12.1% had progression. In 57 patients with available brain tumor assessment, complete or partial intracranial response was reported for 35.7% patients and 5.4% had progression. A total of 17 (3.7%) patients with interstitial lung disease (ILD) was reported with no cases of ILD‐related death.

**Conclusions:**

In this early access program in patients with heavily pretreated HER2‐positive metastatic/unresectable breast cancer, T‐DXd had antitumor activity with a similar response to that reported in previous clinical studies. T‐DXd was well tolerated and no new safety signals were observed.

## 1 INTRODUCTION

The human epidermal growth factor receptor 2 (HER2) is overexpressed in 15%–20% of all breast cancers.^1^, ^2^, ^3^, ^4^, ^5^, ^6^ HER2‐positive breast cancers are more aggressive, but the overexpression of this receptor offers an opportunity for targeted therapy. The introduction of trastuzumab and newer anti‐HER2 drugs has significantly improved the prognosis of patients suffering from HER2‐positive breast cancer. French data from a large‐scale real‐world cohort on metastatic breast cancer reported that median overall survival (OS) was 50.1 (95% CI, 47.6–53.1) months in HER2‐positive patients for the period 2008–2016.^7^ OS dramatically improved in these patients from 2008, after the introduction and extensive use of HER2‐directed therapies (e.g., lapatinib, pertuzumab, trastuzumab emtansine).^8^ Thus, median OS was 39.1 months in 2008 and 58.0 months in 2013. However, durable remission in metastatic breast cancer is rare because none of the available therapies are curative.^9^ In addition, until recently, there was no uniform standard of care for patients with HER2‐positive breast cancer whose disease progressed after two or more HER2‐targeted therapies and options had limited benefit for this patient population.^8^

Trastuzumab deruxtecan (T‐DXd) is an antibody‐drug conjugate (ADC) consisting of an anti‐HER2 antibody (same amino acid sequence than trastuzumab) and a topoisomerase I inhibitor covalently linked by a cleavable tetrapeptide‐based linker.^10^ The DESTINY‐Breast01 trial demonstrated the efficacy of T‐DXd in heavily pretreated HER2‐positive metastatic breast cancer (median of six previous treatments received) in terms of objective response rate (ORR; 62%), median progression‐free survival (PFS; 19.4 months: 95% CI, 14.1–25.0) and median OS (29.1 months: 95% CI, 24.6–36.1).^11^, ^12^ Based on DESTINY‐Breast01 data, T‐DXd received conditional approval from the European Medicines Agency (EMA) on January 18, 2021, as a monotherapy for the treatment of adult patients with unresectable or metastatic HER2‐positive breast cancer who have received at least two prior anti‐HER2‐based therapies.

In France, innovative drugs can be made available to patients who cannot be enrolled in clinical trials prior to Marketing Authorization through a program named Temporary Authorization for Use (ATU). The French Agency for the Safety of Health Products (ANSM) is responsible for delivering ATU, either for a cohort of patients (cohort ATU; cATU) or for a single named patient (nominative ATU; nATU), providing rapid and fair access to innovative drugs. It should be noted that as of July 1, 2021 the various regimes for early access to innovative medicines, including cATU and nATU, have been simplified and replaced by only two authorization regimens: compassionate access (including the former nATU) and early access (including the former cATU). We describe here real‐world data from French patients included in the cATU program with unresectable or metastatic breast cancer treated with T‐DXd after two lines of anti‐HER2‐based regimens in the metastatic setting.

## 2 PATIENTS AND METHODS

### 2.1 Design of early access program and patients

The ANSM granted an early access program for T‐DXd in monotherapy on September 30, 2020. This early access program was intended for the treatment of adult patients with unresectable or metastatic HER2‐positive breast cancer who had received at least two anti‐HER2‐based regimens in the metastatic setting. T‐DXd was already available in the context of nATU since June 30, 2020 and patients who benefited from this treatment were included in the cATU program.

Every cATU access request was evaluated and managed by the Medical Department of Daiichi Sankyo France, in accordance with the Protocol for Therapeutic Use that provided eligibility criteria and patient management information to physicians.

The eligibility criteria were as follows: age > 18 years; unresectable or metastatic breast cancer; diagnosis of HER2‐positive tumor by a validated method (e.g., immunochemistry, IHC; in situ hybridation, ISH); at least two lines of anti‐HER2 therapy in the metastatic setting; normal baseline lung imaging; cardiac examination by ultrasound cardiography or cardiac scintigraphy (MUGA) with LVEF ≥50%; neutrophils ≥1.5 × 10^9^/L. Patients were not eligible in case of history of interstitial lung disease (ILD)/pneumonitis (non‐infectious) requiring corticosteroids or current ILD/pneumonitis; clinically severe lung injury (e.g., pulmonary embolism within 3 months prior to early access program application, severe asthma, severe chronic obstructive pulmonary disease, restrictive lung disease, pleural effusion); autoimmune, connective tissue or inflammatory disorder with pulmonary involvement (e.g., rheumatoid arthritis, Sjögren's syndrome, sarcoidosis); previous pneumonectomy; history of severe hypersensitivity reactions to other monoclonal antibodies; hypersensitivity reactions to drug substances or inactive drug ingredients; pregnant or breastfeeding women.

The recommended dosage for T‐DXd was 5.4 mg/kg administered by intravenous infusion every 3 weeks (21‐day cycle) up to disease progression and/or toxicity. Follow‐up visits and examinations were scheduled in accordance with protocol for therapeutic use. The following examinations were performed during the treatment with T‐DXd: complete blood count at each cycle, chest CT scan every 2 cycles to detect ILD/pneumonitis, cardiac examination (ultrasound or MUGA) every 4 cycles and pregnancy test at each cycle in women of childbearing age.

### 2.2 Data collection

Tumor response measurement was performed in routine practice and radiologically evaluated by the physician. Tumor measurement was recommended every 2 cycles and use of RECIST version 1.1 was preferred. Tumor response data was reported at investigator discretion. The analysis of safety data included adverse drug reactions (ADRs) reported according to the protocol for therapeutic use or spontaneously reported to Daiichi Sankyo France by physicians.

### 2.3 Statistical analysis

The statistical analysis of the data collected during the T‐DXd cATU program was essentially descriptive. Mean values, standard deviations (SD), medians and ranges were calculated for continuous variables whereas counts and percentages were calculated for categorical variables.

## 3 RESULTS

### 3.1 Patient disposition and characteristics

From September 30, 2020 to March 30, 2021, 282 physicians from 155 centers participated in the early access program for T‐DXd in France. A total of 539 applications for early access treatment were received; 49 patients were already treated in the setting of nATU since June 30, 2020 and were included in cATU program. During 6 months, a total of 468 patients were included in the program according to the eligibility criteria; of them 459 were treated. At least one follow‐up and/or discontinuation sheet was collected for 272 patients (59.3% of treated patients). The follow‐up duration was less than 3 months for 62.5% (168/269) and more than 3 months for 37.5% (101/269; 3 missing data).

Among the 459 treated patients, 455 (99.1%) were women with a median age of 58 years (range, 28–92) (Table 1).

**TABLE 1:** Patient characteristics.

	*N* = 459
Age, years, median (range)	58 (28–92)
Weight, kg, median (range)	65 (38–127)
Female gender, *n* (%)	455 (99.1)
Duration from initial diagnosis, months, median (IQR)	79.4 (47.0–133.4)
Metastatic status at initial diagnosis, *n* (%)	
M0: no distant metastasis	274 (59.8)
M1: metastasis to distant organs	180 (39.3)
Mx: unknown	4 (0.9)
Missing	1
HER2+ status, *n* (%)	
IHC 3+	390 (85.0)
IHC 2+/ISH+	64 (13.9)
IHC 2+/ISH−	1 (0.2)
IHC 2+/ISH NA	3 (0.7)
IHC 1+/ISH NA	1 (0.2)
Expression of hormone receptors, *n* (%)	
Positive	290 (67.0)
Negative	143 (33.0)
Missing	26
Tumor status,^a^ *n* (%)	
Unresectable and metastatic	36 (18.9)
Metastatic only	154 (81.1)
Metastatic locations,^a^ *n* (%)	
Bone	263 (57.3)
Lymph nodes	237 (51.6)
Lung	166 (36.2)
Liver	152 (33.1)
Brain	129 (28.1)
Cutaneous/subcutaneous	64 (13.9)
Other	70 (15.3)
ECOG performance status,^a^ *n* (%)	
0	167 (36.4)
1	248 (54.0)
2	42 (9.2)
3	2 (0.4)

Abbreviations: IHC, immunochemistry; ISH, in situ hybridization; NA, not available.

^a^ At the time of early access program application.

At initial diagnosis, 39.3% of patients were found to have de novo metastases (M1); all tumors were considered as HER2‐positive (IHC 3+ for 85% and IHC 2+/ISH+ for 13.9%), except one IHC2+/ISH‐ and four with unknown status (Table 1). At the time of early access program inclusion, the main locations of metastases were bone (57.3%), lymph nodes (51.6%), lung (36.2%), liver (33.1%), and brain (28.1%). Performance status was 0–1 for 90.4% of patients (Table 1).

### 3.2 Prior treatments

A total of 342 (76.5%) patients had undergone surgery and 370 (81.7%) had benefited from radiation therapy (Table 2). All patients had received at least two lines of treatment in the metastatic setting (median number, 4 lines; range, 2–22). Overall, 458 (99.8%) patients had received trastuzumab, 435 (94.8%) trastuzumab emtansine and 364 (79.3%) pertuzumab at metastatic stage, in combination or not (Table 2).

**TABLE 2:** Prior treatments.

	*N* = 459
Prior surgery, *n* (%)	342 (76.5)
Missing	12
Prior radiation therapy, *n* (%)	370 (81.7)
Missing	6
Number of metastatic treatment lines, *n* (%)	
2	97 (21.1)
3	90 (19.6)
4	65 (14.2)
5	67 (14.6)
≥ 6	140 (30.5)
Median (range)	4 (2–22)
Prior treatment for metastatic disease, *n* (%)	
Trastuzumab	458 (99.8)
Trastuzumab emtansine	435 (94.8)
Pertuzumab	364 (79.3)

### 3.3 T‐DXd treatment and follow‐up

For the 459 patients included in the early access program and treated, the planned dosage was 5.4 mg/kg for 452 (98.5%) and 4.4 mg/kg for six patients with the following reasons: liver functions tests (*n* = 2), low weight (*n* = 1), occurrence of adverse event during the treatment in the context of the nominative ATU (*n* = 1), age and chronic myeloid leukemia (*n* = 1) and ECOG performance status at 2 (*n* = 1). The dosage was planned at 3.2 mg/kg for one patient due to poor tolerance of different previous chemotherapies.

For the 272 patients with available follow‐up data, the dose of trastuzumab deruxtecan at initiation was 5.4 mg/kg for 211 patients (98.6%; 58 missing data); 233 (85.7%) patients were being treated at the cut‐off date. For 39 patients, the treatment was discontinued due to the following reasons: disease progression (*n* = 14), death (*n* = 11), patient's wish (*n* = 4), adverse event (*n* = 3), disease progression/death (*n* = 2), disease progression/adverse drug reaction/patient's wish (*n* = 1) and other (*n* = 2) (two missing data). Seventeen interruptions of treatment were reported (12 missing data) and 21 modifications of dosage were reported (11 missing data).

The median duration of T‐DXd treatment was 3.4 (range, 0–7.8) months on March 31, 2021 when the early access program stopped. The early access program was stopped after the release of the commercial treatment in accordance with French drug regulatory rules in the setting of ATU. A total of 233 patients (85.7%) were still being treated at the cut‐off date.

### 3.4 Tumor assessment

Tumor assessment was available in 160 patients. During the early access program, ORR was 56.7% and 12.1% of patients experienced progressive disease (Table 3). Detailed data on patients with partial response, complete response or stable disease were not reported. In 57 patients with available brain tumor assessment, intracranial ORR was 35.7% and 5.4% had progressive disease.

**TABLE 3:** Tumor response during follow‐up.

	*N* = 272^a^
Tumoral assessment, *n* (%)	
Yes	160 (67.2)
No	78 (32.8)
Missing	34
Tumor response, *n* (%)	
Complete or partial response	
Yes	89 (56.7)
No	68 (43.3)
Missing	3
Progression	
Yes	19 (12.1)
No	138 (87.9)
Missing	3

^a^ Patients with at least one follow‐up and/or discontinuation sheet.

### 3.5 Safety and tolerability

During the follow‐up from September 30, 2020 to March 30, 2021, a total of 97 patients out of the 459 treated patients experienced adverse events reported to pharmacovigilance. A total of 41 of these cases were considered as serious. Thirteen fatal cases were reported (related to treatment, *n* = 3; unrelated, *n* = 9; unknown, *n* = 1). The causes of the three treatment‐related deaths were: altered general condition with neurological decompensation and weight loss; lung and cardiac disorders (not ILD); no information was reported on the cause of death of the third case.

These 97 cases reported to pharmacovigilance included 198 adverse drug reactions (ADRs) which are presented in Table 4. The most frequent ADRs were related to gastrointestinal disorders (*n* = 70; 35.4% of all ADRs).

**TABLE 4:** Adverse drug reactions during follow‐up.

System organ class and preferred term	Total ADRs (*n* = 198)	
	Serious^a^	Non‐serious
Gastrointestinal disorders	13 (6.6)	57 (28.8)
Abdominal pain	0	3 (1.5)
Constipation	0	1 (0.5)
Diarrhea	3 (1.5)	4 (2.0)
Dyspepsia	0	1 (0.5)
Gastritis	0	2 (1.0)
Gastrointestinal disorder	1 (0.5)	1 (0.5)
Gastrointestinal reflux disease	0	1 (0.5)
Nausea	5 (2.5)	35 (17.7)
Vomiting	4 (2.0)	9 (4.5)
General disorders and administration site conditions	11 (5.6)	22 (11.1)
Asthenia	4 (2.0)	20 (10.1)
Chest discomfort	1 (0.5)	0
Condition aggravated	1 (0.5)	0
Death	1 (0.5)	0
Device‐related thrombosis	1 (0.5)	0
General physical health deterioration	1 (0.5)	0
Pyrexia	0	2 (1.0)
Respiratory, thoracic and mediastinal disorders	12 (6.1)	11 (5.6)
Cough	0	2 (1.0)
Dyspnea	1 (0.5)	2 (1.0)
Interstitial lung disease	9 (4.5)	5 (2.5)
Lung disorder	2 (1.0)	2 (1.0)
Blood and lymphatic systems disorders	2 (1.0)	17 (8.6)
Anemia	1 (0.5)	7 (3.5)
Bone marrow failure	1 (0.5)	0
Neutropenia	0	9 (4.5)
Thrombocytopenia	0	1 (0.5)
Infections and infestations	9 (4.5)	0
COVID‐19	3 (1.5)	0
*Escherichia. coli* bacteriemia	1 (0.5)	0
*Escherichia coli* sepsis	1 (0.5)	0
Ophthalmic herpes zoster	1 (0.5)	0
*Pneumocystis jirovecii* pneumonia	2 (1.0)	0
Superinfection	1 (0.5)	0
Investigations	1 (0.5)	6 (3.0)
General physical condition abnormal	1 (0.5)	0
Ejection fraction decreased	0	1 (0.5)
Hepatic enzyme abnormal	0	1 (0.5)
Liver function test increased	0	1 (0.5)
Weight decreased	0	3 (1.5)
Nervous system disorders	4 (2.0)	7 (3.5)
Dysgeusia	0	2 (1.0)
Dizziness	0	1 (0.5)
Headache	0	4 (2.0)
Intracranial pressure increased	1 (0.5)	0
Meningorrhagia	1 (0.5)	0
Neurological decompensation	1 (0.5)	0
Seizure	1 (0.5)	0
Hepatobiliary disorders	3 (1.5)	3 (1.5)
Cholestasis	1 (0.5)	1 (0.5)
Hepatocellular injury	2 (1.0)	1 (0.5)
Hypertransaminasemia	0	1 (0.5)
Skin and subcutaneous tissue disorders	1 (0.5)	5 (2.5)
Alopecia	0	5 (2.5)
Rash	1 (0.5)	0
Metabolism and nutrition disorders	1 (0.5)	7 (3.5)
Decreased appetite	1 (0.5)	5 (2.5)
Feeding disorder	0	1 (0.5)
Hypokalemia	0	1 (0.5)
Neoplasms (benign, malignant and unspecified)	1 (0.5)	1 (0.5)
Hemangioma	1 (0.5)	0
Liver metastases	0	1 (0.5)
Cardiac disorder	1 (0.5)	0
Cardiac disorder	1 (0.5)	0
Ear and labyrinth disorders	0	1 (0.5)
Vertigo	0	1 (0.5)
Eye disorders	0	1 (0.5)
Diplopia	0	1 (0.5)
Musculoskeletal and connective tissue disorders	1 (0.5)	0
Muscular weakness	1 (0.5)	0

*Note*: Results are presented as *n* (%).

^a^ Defined as an adverse reaction that results in death, is life‐threatening, requires hospitalization or prolongation of existing hospitalization, results in persistent or significant disability or incapacity, or is a birth defect.

ILD was initially reported for 14 patients by physicians. A total of 17 cases (3.7%) were identified as ILD or considered ILD retrospectively. There was no case of ILD with fatal outcome (only grades 1–3 were reported by physicians).

## 4 DISCUSSION

HER2‐positive metastatic breast cancer patients included in this cATU program received at least two prior lines of anti‐HER2 in the metastatic setting and were heavily pretreated with a median of 4 (range, 2–22) metastatic treatment lines. At treatment initiation, 98.6% of patients received T‐DXd at 5.4 mg/kg. These data therefore indicate that the eligibility criteria for inclusion in the early access program cohort were met and that patients were treated as recommended.

The median age of patients in the cATU program (58 years) was comparable to the one reported in the DESTINY‐Breast studies (55 years in DESTINY‐Breast01 and 54.2 years in DESTINY‐Breast02).^11^, ^13^ The DESTINY‐Breast studies included patients in good general condition and > 99% patients had a performance status at 0–1; in contrast, 9.6% of patients in the cATU program had a performance status ≥2. In addition, the DESTINY‐Breast studies excluded patients with symptomatic or progressive brain metastases. In the cATU program, patients were not excluded on this criterion (28.1% had brain metastases active or stable). As expected for a real‐world cohort, these data indicate that the patients included in the cATU program were in poorer condition compared to patients in the DESTINY‐Breast trials.

In the ESME cohort, a large national observational database, patients of the HER2+ subgroup had a median age of 57 years at diagnosis of metastatic disease, 83% had performance status 0–1, 21.5% had ≥3 metastatic sites and 11.3% had brain metastases.^7^ Thus, patients with HER2‐positive metastatic breast cancer in the ESME cohort had similar characteristics to those in the present cohort.

During the 6 month follow‐up of the cATU program, ORR was 56.7% in patients with available data. In addition, data on brain metastases were reported; for patients with available data, ORR was 35.7%. In the phase II DESTINY‐Breast01 study, which included heavily pretreated patients (median, six lines) with HER2‐positive metastatic breast cancer, ORR was 60.9% (95% CI 53.4–68.0), including 6.0% of complete response and 54.9% of partial response; disease control rate was 97.3% (95% CI, 93.8–99.1).^11^ In an update after a median follow‐up of 26.5 months, ORR was 62.2%.^12^ The phase III DESTINY‐Breast02 study was the confirmatory study of the DESTINY‐Breast01 study; it included HER2‐positive unresectable or metastatic breast cancer previously treated with trastuzumab emtansine.^13^ Patients received a median of two previous line of therapy in the setting of metastatic disease. ORR was 69.7% (95 CI, 65.0–74.1) in patients treated with T‐DXd including 14.0% with a complete response.

Safety profile of T‐DXd in cATU program was manageable and no new safety signals were observed. The rates of adverse events reported in the cATU program were lower than those reported in trials. An important factor to consider for the report of adverse events in our cohort, especially for ILD, is the short duration of follow‐up (<3 months for 62.5% of patients) compared to the DESTINY studies (median of 11.1 months in DESTINY‐Breast01, 21.5 months in DESTINY‐Breast02 and 28.4 months in DESTINY‐Breast03).^11^, ^13^, ^14^ The most frequent adverse events were gastrointestinal, mostly nausea. A total of 17 cases (3.7%) of ILD were reported or considered ILD retrospectively, but no fatal cases related to ILD were reported. ILD is an important identified risk in the Risk management plan of T‐DXd. T‐DXd's Summary of product characteristics recommends permanent discontinuation in case of ILD of Grade 2 and higher.^15^ For Grade 1 ILD (no symptoms, only radiologic evidence), T‐DXd may be resumed after complete resolution (dose is reduced if resolution requires more than 28 days); for Grade 2 ILD, permanent discontinuation is recommended. A close follow‐up of patients (early report of symptoms, CT‐scan) is essential to prevent high‐grade ILD. The rates of ILD were 13.6%, 10.4%, and 15.2% in the DESTINY‐Breast01, ‐Breast02 and ‐Breast03 studies, respectively.^11^, ^13^, ^14^

Among the strengths of the analysis of this cATU program is the large number of patients and centers. In addition, this real‐world population corresponded to T‐DXd therapeutic indication and expected use in routine clinical practice and not a population meeting the stringent inclusion/exclusion criteria of clinical trials. In particular, patients with active brain metastases were not excluded. Of note, the fast enrollment of 468 patients in a short period of time illustrates the unmet medical need for this highly pretreated population.

The limitations of the analysis of the cATU program are those of an observational study. There was no centralized tumor assessment and data from physicians enrolling patients were only declarative. The number of missing data for efficacy was large and comparisons with clinical studies are inappropriate. In addition, some data are currently unavailable (e.g., time of onset of ILD, percentage of active/stable metastases). The short follow‐up duration is also an important limitation. Indeed, the early access program started on September 30, 2020 and ended on March 30, 2021 with the release of the commercial treatment.

Patients of T‐DXd cATU program had the opportunity to be included in the REALITY‐01 study, a French non‐interventional study aimed to collect data on long‐term safety of T‐DXd. As of December 1, 2022, 306 patients have been enrolled in REALITY‐01, including 113 patients from the T‐DXd cATU program (unknown initial cohort for eight patients of REALITY‐01). Data on patient profile, treatment history, effectiveness, and health‐related quality of life will also be collected to complement data from clinical trials. Based on the DESTINY‐Breast03 results, T‐DXd has been recently approved by the Committee for Medicinal Products for Human Use (CHMP) of the European Medicines Agency (EMA) as second‐line treatment of patients with metastatic breast cancer HER2‐positive previously treated with an anti‐HER2‐based regimen. The French Agency for the Safety of Health Products (ANSM) granted an early access program for T‐DXd as a single agent in this indication. In addition, recently published results from the DESTINY‐Breast04 trial offer new treatment perspectives for patients with low HER2 metastatic breast cancer.^16^

In conclusion, this cATU program reports real‐world data of T‐DXd in patients with heavily pretreated HER2‐positive metastatic/unresectable breast cancer. T‐DXd had antitumor activity with a similar response to that reported in previous clinical studies and was well tolerated. No new safety signals were reported.

## AUTHOR CONTRIBUTIONS

**Thierry Petit:** Conceptualization (equal); data curation (equal); writing – original draft (equal); writing – review and editing (equal). **Nawale Hajjaji:** Conceptualization (equal); data curation (equal); validation (equal); visualization (equal); writing – review and editing (equal). **Eric‐Charles Antoine:** Conceptualization (equal); writing – review and editing (equal). **Marc‐Antoine Benderra:** Conceptualization (equal); writing – review and editing (equal). **Michel Gozy:** Conceptualization (equal); writing – original draft (equal). **Cyril Foa:** Validation (equal); visualization (equal); writing – review and editing (equal). **Jean‐Loup Mouysset:** Validation (equal); writing – review and editing (equal). **Julien Grenier:** Investigation (equal); validation (equal); visualization (equal); writing – review and editing (equal). **Mireille Mousseau:** Conceptualization (equal); data curation (equal); formal analysis (equal); funding acquisition (equal); investigation (equal); methodology (equal); project administration (equal); supervision (equal); validation (equal); visualization (equal); writing – review and editing (equal). **Audrey Mailliez:** Conceptualization (equal); writing – review and editing (equal). **Mahasti Saghatchian:** Investigation (equal); resources (equal); validation (equal); visualization (equal); writing – review and editing (equal). **Emma Lachaier:** Data curation (equal); investigation (equal); visualization (equal); writing – review and editing (equal). **Isabelle Desmoulins:** Conceptualization (equal); validation (equal); visualization (equal); writing – review and editing (equal). **Audrey Hennequin:** Validation (equal); visualization (equal); writing – review and editing (equal). **Patricia Maes:** Conceptualization (equal); writing – review and editing (equal). **Delphine Loirat:** Conceptualization (equal); writing – review and editing (equal). **Francesco Ricci:** Data curation (equal); investigation (equal); methodology (equal); validation (equal); writing – review and editing (equal). **Véronique Diéras:** Conceptualization (equal); data curation (equal); validation (equal); visualization (equal); writing – review and editing (equal). **Dominique Berton:** Conceptualization (equal); writing – review and editing (equal). **Florence Lai Tiong:** Conceptualization (equal); writing – review and editing (equal). **Luis Teixeira:** Conceptualization (equal); investigation (equal); validation (equal); writing – review and editing (equal). **Nadine Dohollou:** Conceptualization (equal); data curation (equal); validation (equal); visualization (equal); writing – review and editing (equal). **Christelle Lévy:** Conceptualization (equal); writing – review and editing (equal). **Thomas Bachelot:** Conceptualization (equal); writing – review and editing (equal). **Jean‐Yves Pierga:** Conceptualization (equal); investigation (equal); resources (equal); validation (equal); writing – review and editing (equal).

## FUNDING INFORMATION

AstraZeneca and Daiichi Sankyo.

## CONFLICT OF INTEREST STATEMENT

Nawale Hajjaji reports competing interests with Pfizer, Novartis, Lilly, Daiichi Sankyo, Gilead, AstraZeneca (consulting fees), Novartis, Pfizer (research grant to institution), and Daiichi Sankyo, AstraZeneca (travel expenses); Marc‐Antoine Benderra reports competing interest with Daiichi Sankyo support for attending meetings and/or travel; participation on a Data Safety Monitoring Board or Advisory Board; Cyril Foa reports competing interests with Roche, MSD, AstraZeneca, Novartis; Julien Grenier reports competing interest for Daiichi Sankyo (congress) and AstraZeneca/Daiichi Sankyo (board); Mahasti Saghatchian reports competing interests with AstraZeneca (consulting/expert, speaker/conferences, research grants/clinical trials), BMS (research grants/clinical trials), Daiichi Sankyo (consulting/expert, speaker/conferences, research grants/clinical trials, conference travel), Eisai (consulting/expert, speaker/conferences), Myriad Genetics (consulting/expert, speaker/conferences, research grants/clinical trials), Seagen (consulting/expert, speaker/conferences, research grants/clinical trials, conference travel), MSD (consulting/expert, research grants/clinical trials, conference travel), Novartis (consulting/expert, speaker/conferences, research grants/clinical trials, conference travel), Pfizer (consulting/expert, speaker/conferences, research grants/clinical trials, conference travel), Viatris (consulting/expert, speaker/conferences), Roche (consulting/expert, speaker/conferences, research grants/clinical trials, conference travel), Menarini‐Stemline (consulting/expert); Véronique Diéras reports competing interest with Roche, Novartis, Pfizer, Lilly, AstraZeneca, Daiichi Sankyo, Seagen, Gilead, MSD (travel expenses), Roche/Genentech, Novartis, Lilly, Pfizer, AstraZeneca, AbbVie, MSD, Daiichi Sankyo, Seagen, Gilead, Eisai, Pierre Fabre Oncologie, Medac GmbH, Menarini (honoraria for consulting), Roche, Novartis, Pfizer, Lilly, Astra Zeneca, Daiichi Sankyo, Seagen, Gilead (honoraria for symposia); Luis Teixeira reports competing interest with Roche, Novartis, AZD, Daiichi‐Sankyo (consulting or advisory role); Nadine Dohollou reports competing interests with AstraZeneca, BMS, Boehringer Ingelheim, Genomic Health, Lilly, MSD, Novartis, Pfizer, Roche (research grants for clinical trials), Daiichi, Lilly, Roche, Seagen (consulting/expert and conferences/trainings); Thomas Bachelot reports personal fees (Seagen), grants to institution (Seagen), personal fees (Pfizer), grants to institution (Pfizer), personal fees (AstraZeneca/Daiichi), grants to institution (AstraZeneca/Daiichi), personal fees (Novartis), grants to institution (Novartis), personal fees (Lilly), non‐financial support (Pfizer). The other authors did not report competing interests.

## ETHICS STATEMENT

Patients' information to access the T‐DXd cATU program: The T‐DXd cATU program was conducted in accordance with the Declaration of Helsinki. An ATU is a compassionate early access program allowing for advanced use of a medication that has not yet received marketing authorization. According to specific regulations governing French early access programs, no ethical approval from institutional review board or ethics committee and no written consent were required. Patients were fully informed of their data privacy rights and received written information. French Health Authorities approved and validated the therapeutic use and real‐life data collection in patients receiving T‐DXd which was defined in a Temporary Use and Data Collection Protocol (PUT‐RD). Patients' non‐opposition to the research using the data of the T‐DXd cATU program: The research study complied with French regulations. Patients received a written information explaining the purpose of the research and each patient provided a non‐opposition. The study was approved by an Independent Ethics Committee on 28/10/2021 (2021‐A02007‐34) and complied with CNIL requirements (« Commission nationale de l'informatique et des libertés »).

## Data Availability

The datasets used and analyzed during the current study are available from the corresponding author on reasonable request.
